# Clinicopathological Features and Survival of Signet-Ring Cell Carcinoma and Mucinous Adenocarcinoma of Right Colon, Left Colon, and Rectum

**DOI:** 10.3389/pore.2021.1609800

**Published:** 2021-07-02

**Authors:** Lili Zhu, Chunrun Ling, Tao Xu, Jinglin Zhang, Yujie Zhang, Yingjie Liu, Chao Fang, Lie Yang, Wen Zhuang, Rui Wang, Jie Ping, Mojin Wang

**Affiliations:** ^1^ Department of Gastrointestinal Surgery, West China Hospital, Sichuan University, Chengdu, China; ^2^ Department of General and Pediatric Surgery, The People’s Hospital of Guangxi Zhuang Autonomous Region, Nanning, China; ^3^ Department of General Surgery, Suining Municipal Hospital of Traditional Chinese Medicine, Suining, China; ^4^ Department of Gastrointestinal Surgery, Yibin Second People’s Hospital, Yibin, China; ^5^ Department of Gastroenterology, West China Hospital, Sichuan University, Chengdu, China; ^6^ Division of Epidemiology, Vanderbilt University Medical Center, Nashville, TN, United States

**Keywords:** survival, colorectal carcinoma, signet-ring cell carcinoma, histological subtype, SEER

## Abstract

Histological subtype plays an important role in the different clinical characteristics and survival outcomes of patients with colorectal carcinoma (CRC). However, in previous studies, the influences of tumor locations and tumor stages have not been strictly controlled. This study focused on the assessment of the prognostic value of each histological subtype in different tumor locations and tumor stages of CRC. We used the Surveillance, Epidemiology, and End Results (SEER) database (1973–2011) to analyze 818,229 CRC patients with different clinical and pathological features, and analyzed the prognostic value of each histological subtype. Under the condition of stratification by tumor stage, signet-ring cell carcinoma (SRCC) presented the worst survival in each stage of right colon cancer (stage I, log-rank, *p* = 0.002, stages II, III, and IV, log-rank, *p* < 0.001), rectal cancer (RC) (log-rank, *p* < 0.001), and in stages II, III, and IV of left colon cancer (log-rank, *p* < 0.001). Multivariate survival analysis suggested SRCC subtype, male gender, age ≥ 70 years, tumor size ≥ 5 cm, stage progression, and poor differentiation were all significant factors worsening survival in CRC (*p* < 0.001, respectively). Mucinous adenocarcinoma (MC) histological subtype proved to be an independent protective factor for the prognosis of right colon cancer (*p* = 0.003). Overall, in our study, the results suggested SRCC had the worst survival among the three histological subtypes of CRC. MC was associated with favorable prognosis in right colon cancer but not with other tumor locations.

## Introduction

Colorectal carcinoma (CRC) represents one of the most common health burdens worldwide. The histological classification of CRCs was according to the World Health Organization classification system. Non-mucinous adenocarcinoma (NMC) is the most common histological subtype, followed by mucinous adenocarcinoma (MC), and signet-ring cell carcinoma (SRCC). In contrast to NMC, MC and SRCC are adenocarcinomas in which the cancer cells produce excess mucin. MC accounts for 10–15% of CRC and is composed of extracellular mucin pools that cover more than 50% of the lesion [1,2]. SRCC, which accounts for 1% of CRC, is defined as signet-ring cells making up more than 50% of the tumor area. In addition, CRC that contains both mucinous and signet-ring cell components is currently classified as MC if extracellular mucins make up more than 50% of the tumor [1,2]. The histological subtype is considered to play a role in CRC patients with different clinical characteristics and survival outcomes. Though the prognosis of MC is still controversial [3–9], SRCC proved to be associated with impaired outcome in almost all previous studies [10–18]. Because tumor location and tumor stage have been related to the prognosis, these variables could have acted as potential confounders in previous researches. To assess a prognostic role of histological subtype independent of tumor location and stage, it is necessary to control these key factors strictly.

In this study, we analyzed the prognostic value of histological subtype in CRC, using the data from the Surveillance, Epidemiology, and End Results (SEER) database, which included a large number of patients from the United States national population-based data. We hypothesized that the prognostic value of each histological subtype might differ in different tumor locations and tumor stages of CRC.

## Patients and Methods

### Surveillance, Epidemiology, and End Results Database

The data of 818,229 CRC patients with different clinical and pathological characteristics were retrieved from the SEER 18 database (1973–2011), released on April 2014 (November 2013 submission). The SEER database collected and published patients from population-based cancer registries comprising approximately 27.8% of the United States Population (http://seer.cancer.gov/about/overview.html). Records of each patient’s features included demographics (diagnosis of age, race, gender), year of diagnosis, tumor characteristics (location, number, size, TNM stage, tumor grade, histological type, and numbers of lymph nodes examined), and treatments [cancer-directed surgery (CDS), radiotherapy].

### Patients Selection

Patients with primary CRC (C20.9) were selected for this study according to the International Classification of Diseases for Oncology (third edition, ICD-O-3) codes. Data from eligible patients were stratified by different histological subtypes in different tumor locations and tumor stages of CRC.

### Data Analysis

According to the ICD-O-3, the tumor histological subtypes were classified as SRCC (8,490), MC (8,480, 8,481) and NMC (8,010, 8,140–8,141, 8,144–8,145, 8,210–8,211, 8,220–8,221, 8,230–8,231, 8,260–8,263). Based on the criteria described in the American Joint Committee on *Cancer* (AJCC) *Cancer* Staging Manual, seventh edition, 2010, all TNM classifications were restaged, and histological grades were divided into well differentiated (G1), moderately differentiated (G2), poorly differentiated (G3), and undifferentiated (G4). The 5-year cancer specific survival (CSS) rates and CRC-SS time were calculated from the time of diagnosis to the time of cancer-specific death or the end of follow-up (cutoff date: December 2011). Deaths from CRC were regarded as events, while deaths attributed to other causes were considered to be censored observation.

### Statistical Analysis

All statistical analyses were performed with the R version 3.1.2 (http://www.r-project.org/). Clinicopathological characteristics of different histological subtypes were analyzed by independent t-test and Chi-square test. Continuous data was expressed as mean ± standard deviation. The Kaplan-Meier method was used for generating survival curves. The log-rank test was used to analyze the difference between the curves. Multivariate Cox proportional hazard regression models were built to analyze the impact of the analyzed factors. The data was summarized with hazard ratios (HR) and 95% confidence intervals (CI). All statistical tests were 2-sided, and a *p* value of no more than 0.05 was judged as statistically significant.

## Results

### The Demographics of Colorectal Carcinoma Patients

A total of 818,229 patients with CRC were selected for this study. The clinical and pathological characteristics of CRC patients with different pathological subtypes are shown in Supplementary Table S1. 741,207 cases were diagnosed with NMC (90.6%), 70,555 (8.6%) with MC, and 6,467 (0.8%) with SRCC, respectively.

### The Proportion of Signet-Ring Cell Carcinoma in Colorectal Carcinoma in Time Course

Change of the proportion was obviously observed in the SRCC subtype. The total percentage of colorectal SRCC rose to 1.02% in the period of 2001–2011, up from 0.25% in 1973–1990 and 0.88% in 1991–2000. The frequency of SRCC in the right colon was much higher than in the left colon and the rectum in each period (0.33% vs. 0.19% and 0.23%, 1973–1990; 1.32% vs. 0.51% and 0.66%, 1991–2000; 1.41% vs. 0.63% and 0.81%, 2001–2011) (Supplementary Table S2).

### Clinicopathological Characteristics of Signet-Ring Cell Carcinoma in Colorectal Carcinoma Patients

The clinicopathological features of MC, NMC, and SRCC patients are shown in Supplementary Table S1. There were higher percentages of males in SRCC and NMC (vs. MC, *p* < 0.001). Compared with MC and NMC, SRCC patients showed significant differences in clinicopathological characteristics: age at diagnosis (younger, *p* < 0.001), tumor numbers (more patients with single tumor, *p* < 0.001), TNM stage (more advanced in stage, *p* < 0.001), tumor grade (poorer differentiation, *p* < 0.001), CDS (lower numbers were performed, *p* < 0.001), radiotherapy (more patients be assigned for radiotherapy, *p* < 0.001). Then compared with NMC, SRCC, and MC were more likely to occur in the right colon, more frequently with larger tumor size (> 5 cm) and with higher numbers of examined lymph nodes (NO ≥ 12) (*p* < 0.001).

### Survival in Colorectal Signet-Ring Cell Carcinoma, Mucinous Adenocarcinoma, and Non-mucinous Adenocarcinoma Patients

**FIGURE 1:**
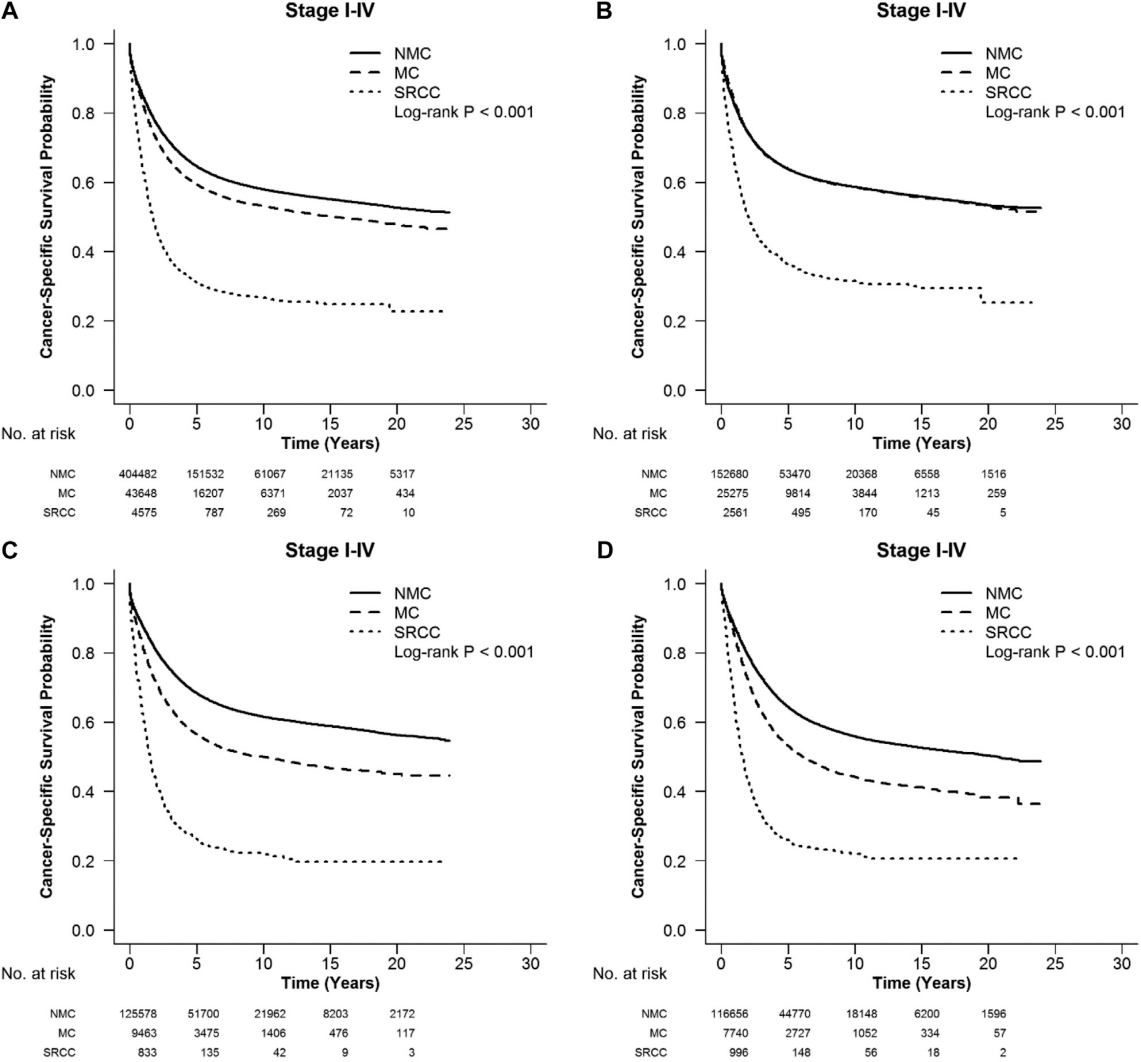
CSS with stages I-IV colorectal SRCC, MC, and NMC at different locations: **(A)** whole colon, **(B)** right colon, **(C)** left colon and **(D)** rectum. Compared with MC and NMC patients, SRCC patients had a significantly worse CSS among different locations of CRC (log-rank, *p* < 0.001).

**FIGURE 2:**
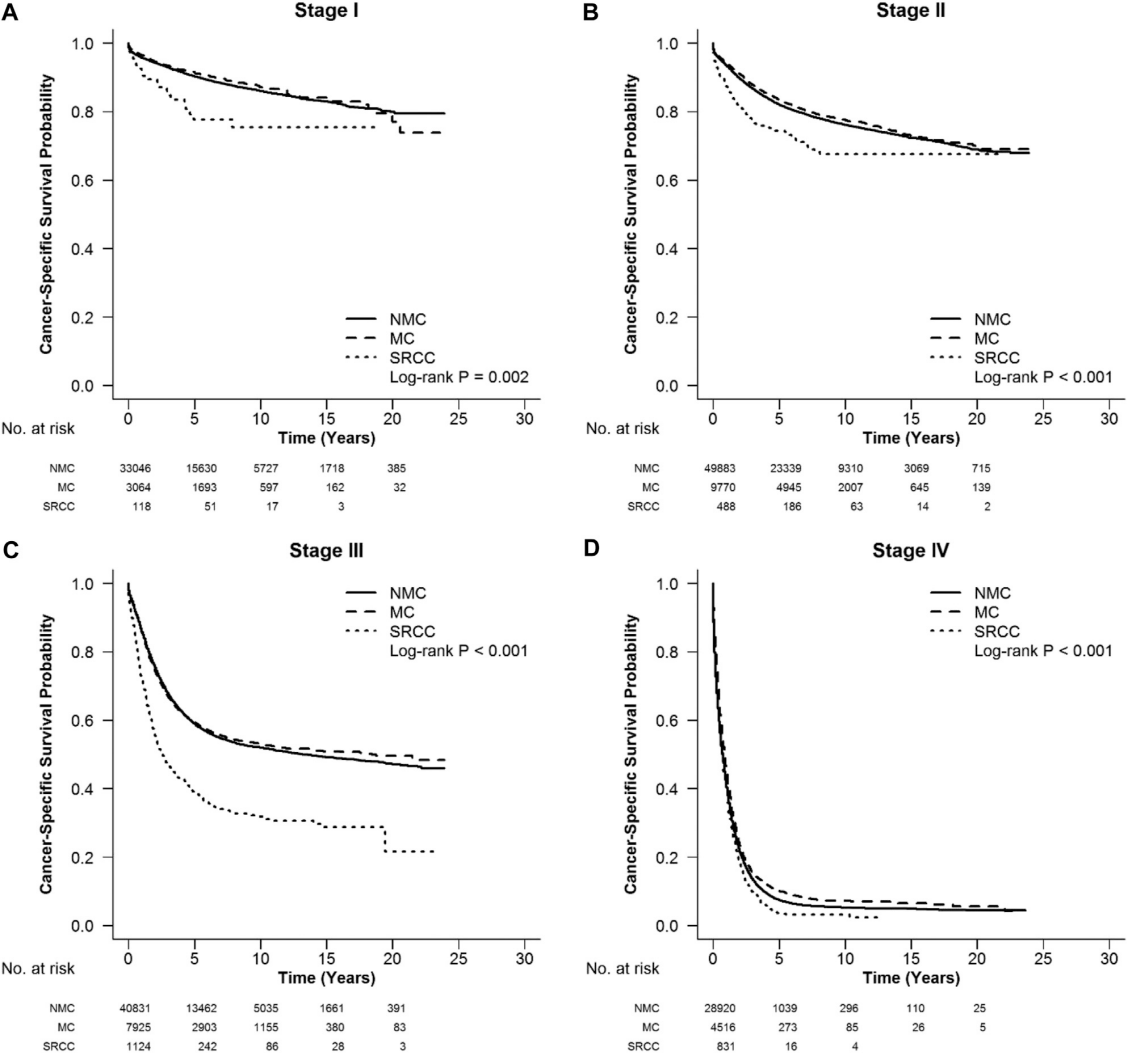
CSS in different stages of right colon cancer among SRCC, MC, and NMC patients **(A–D)** SRCC patients had the worst CSS in each stage of right colon cancer, while MC patients presented better survival [**(A)**, log-rank, *p* = 0.002, **(B–D)**, log-rank, *p* < 0.001].

**FIGURE 3:**
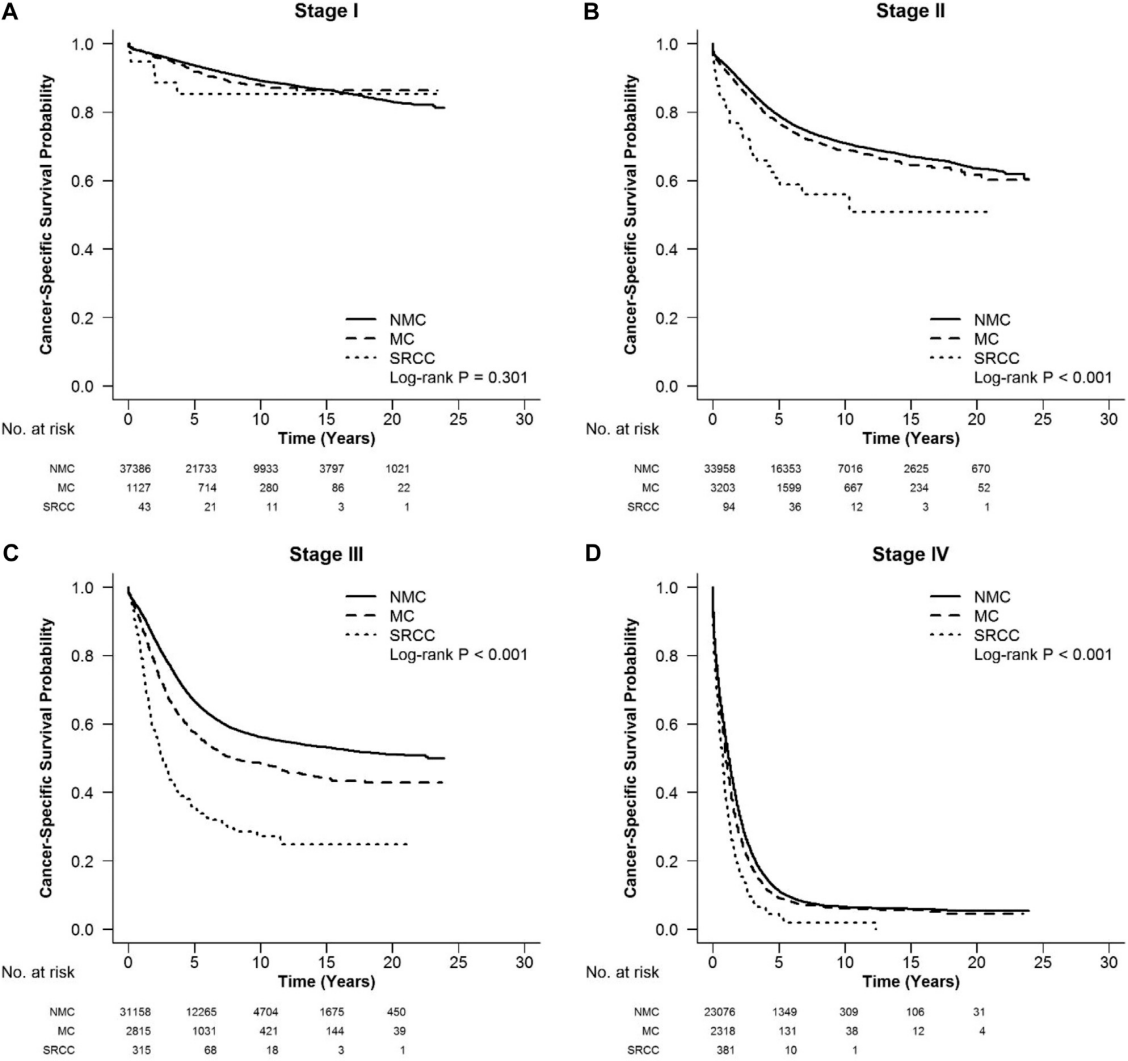
CSS in different stages of left colon cancer among SRCC, MC, and NMC patients **(A)** No significant difference among the survival of three subtypes in stage I of left colon cancer (log-rank, *p* = 0.301). **(B–D)** SRCC patients presented the worst CSS in stages II, III, and IV of left colon cancer (log-rank, *p* < 0.001).

**FIGURE 4:**
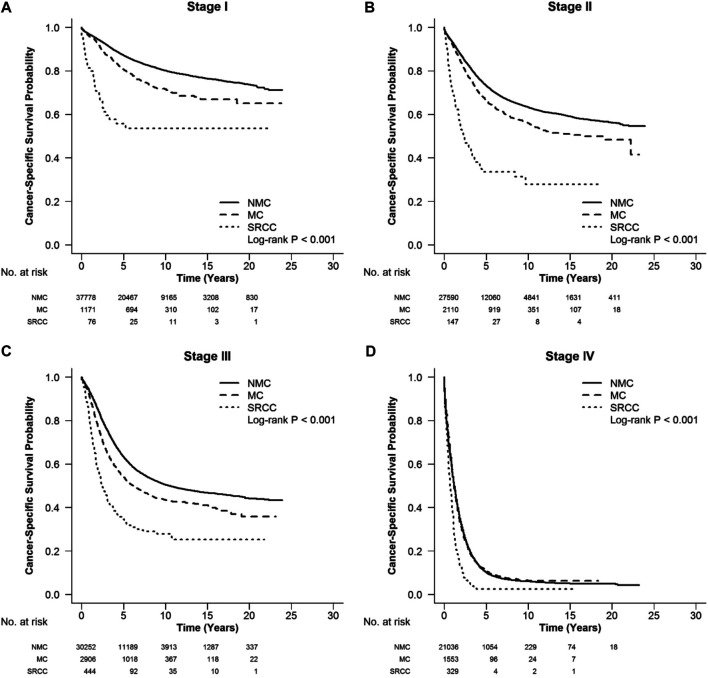
CSS in different stages of RC among SRCC, MC and NMC patients **(A–D)** SRCC presented the worst CSS in each stage of RC (log-rank, *p* < 0.001).

According to the CSS curve, SRCC patients had a worse prognosis compared with MC and NMC patients (Figures 1A−D, log-rank, *p* < 0.001). When stratified by tumor stage, SRCC presented the worst survival in each stage of right colon cancer and rectal cancer (RC), and stages II, III, and IV of left colon cancer (Figure 2A log-rank, *p* = 0.002, Figures 2B−D, 3B−D, 3B−D, 4A−D log-rank, *p* < 0.001, all). There was no significant difference among the survival of the three subtypes in stage I of left colon cancer (Figure 3A, log-rank, *p* = 0.301). Compared with NMC and SRCC, MC patients had better survival in each stage of right colon cancer (Figures 2A−D, log-rank, *p* < 0.001, all). In terms of 5-year survival (Supplementary Table S3), there was no significant difference between MC and SRCC or MC and NMC in stage I of left colon cancer (MC vs. SRCC, *p* = 0.141 and MC vs. NMC, *p* = 0.056), but SRCC was associated with worse survival than NMC (stage I, *p* = 0.042, stages II, III, and IV, *p* < 0.001). Although NMC and MC patients had better 5-year survival than SRCC patients (*p* < 0.001), no significant difference could be found between the two subtypes in stages I and III of right colon cancer (NMC vs. MC, *p* = 0.088, 0.996, respectively), and stage IV of RC (NMC vs. MC, *p* = 0.757).

### Multivariate Analysis of Prognostic Factors in Colorectal Carcinoma Patients

The results of multivariate survival analysis for all CRC patients using Cox model are shown in Supplementary Table S4. According to this analysis, SRCC subtype, male gender, age ≥ 70 years, tumor size > 5 cm, advanced a higher stage, poor differentiation were all significant factors in the deterioration of CRC survival (*p* < 0.001, all). In contrast, multiple tumors, accepted CDS and a greater number of lymph nodes detected (≥ 12) were associated with better survival (*p* < 0.001, all). Notably, MC histological subtype proved to be an independent protective factor of prognosis in the right colon (*p* = 0.003), but not in the left colon or rectum. Radiotherapy proved to be an independent protective factor of RC.

## Discussion

In most of the relating studies, mucin-secreting adenocarcinoma (including MC and SRCC) and right-sided colon cancer were associated with adverse prognosis [10–12,19]. The occurrence of these histological subtypes may differ widely among different geographic areas. For SRCC, according to the literatures, the frequency could vary from 1% in the United States to 18.5% in Jordan and Lebanon [19,20]. In this study, the proportion of colorectal SRCC in the SEER database has been rising during the past decades. The frequency of SRCC in the right colon was much higher than in the left colon and the rectum in each period, which was consistent with previous reports. In these studies, right-sided colon cancer was considered to have something to do with worse prognosis compared with the left-sided colon cancer and RC [16]. The higher frequency of SRCC in right-sided colon cancer may also contribute to the poorer outcome of this group.

This study confirmed a number of recognized characteristics associated with SRCC, such as younger age at diagnosis, more patients with single tumor, more advanced tumors, and poorer tumor grade. Additionally, our research showed that colorectal SRCC patients were less likely to achieve a CDS and more often be assigned for radiotherapy than MC and NMC in RC. Consistent with the study of Vallam et al. [21], in which patients with SRCC had a higher rate of circumferential resection margin (CRM) positivity (19%) than patients with non-SRCC (4%), making a CDS impossible. In a previous study concerning RC [22], we also observed that the patients in SRCC group were more likely to receive preoperative radiotherapy (PRT) compared with patients in the MC and NMC groups, but SRCC remained a factor of poor prognosis in locally advanced stage (stages II and III) RC patients who were in a PRT setting. Both phenomena might be attributed to advanced stage at diagnosis for this special entity. Besides, a predilection to occur in the right colon, larger tumor size, and a greater number of lymph nodes examined were common features between SRCC and MC.

Although colorectal SRCC and MC were considered to be associated with poor prognosis in many previous studies, reports on the prognosis value of SRCC and MC stratified by tumor location and stage are still rare. From our results, SRCC had the worst CSS in all patients and at each stage of each tumor location, except for stage I of left-sided colon cancer. One of the reasons for the poor prognosis of SRCC may be advanced stage at diagnosis, as mentioned above. The higher CRM-positive rate in SRCC made a CDS impossible, leading to a higher risk of local recurrence and worse survival [10,21,23,24]. Low differentiation at diagnosis, associated with high risk of vascular invasion and lymph node involvement [17,24], may be another explanation for the adverse prognosis of SRCC. Furthermore, in their study, Ho-Su and colleagues [25] presented a higher incidence of scirrhous carcinoma in the SRCC group than the NMC group, which might lead to symptoms of intestinal obstruction. Bowel obstruction may prevent the endoscope to pass beyond the tumor, and finally can affect the rate of missed diagnosis of synchronous carcinoma in colorectal SRCC. Besides, in the same research, they found that most patients with SRCC showed an invasive growth pattern, which had been shown to be an independent prognostic factor in patients with stages I-III CRC. In other studies [11,23], the decreased expression of adhesion molecules (such as E-cadherin, β-catenin) and the amplification of Bcl-224 were related to reduce cell-cell adhesion, loosen the surrounding structure of tumor cells and enabled them to spread far away or form peritoneal metastases, which could not be treated by radical surgery and lead to poor prognosis.

Though MC has been reported to have some similar clinicopathological features compared with SRCC, the prognostic value of MC is still controversial [3–9]. Hugen et al. [6] reported that the poor prognosis for MC was only seen in RC. Gao et al. [4] found that MC was associated independently with poorer prognosis for RC and was an independent protective survival indicator in right-sided colon cancer. In our study, MC acted as a favorable factor in right-sided colon cancer, in keeping with Peng’s research. The mechanism could potentially be related to the interaction of MC with other site-specific factors. One of the hypotheses is that hereditary non-polyposis colorectal cancer (HNPCC) and high-frequency microsatellite instability (MSI-H) tend to be associated with right-sided of colon cancer, and usually occur in MC. HNPCC and MSI-H have proven to be associated with good response to adjuvant therapy and improved disease free survival [26–29]. Therefore, the protective effect of MC in right-sided colon cancer may be due to the higher proportion of HNPCC and MSI-H in this tumor location group. In a study of colorectal MC, Chung-Ta et al. [30] found that MCs without both serrated adenocarcinoma (SAC) morphology and CpG island methylator phenotype (CIMP) positive status exhibited 3.955 times greater risk of cancer relapse than MCs having both characteristics or either one, and SAC was associated with proximal location and CIMP-positive status, which may be another explanation for the phenomenon. Despite all these, further research is still needed to confirm this phenomenon and elucidate the mechanism behind it.

In the 5-year survival analysis, there were no differences between MC and NMC in stages I and III of right colon, stage I of left colon, and stage IV of rectum. The intrinsic mechanism to explain this phenomenon has not been reported.

In consistency with previous findings, the current study also showed that SRCC subtype, male gender, age ≥ 70 years, tumor size > 5 cm, advanced stage, poor differentiation were all significant factors in the deterioration of CRC survival. In contrast, multiple tumor numbers, accepted CDS, higher numbers of examined lymph nodes (≥ 12), administration of radiotherapy were associated with better survival. Notably, MC histological subtype proved to be an independent protective factor of prognosis in right colon, but when it appeared in left colon or rectum, an opposite effect was observed. Our findings suggested that clinical decision should take these factors into account.

Our study data included a large number of patients from the United States, using national population-based data, avoiding the biases associated with single-institution experience or limited sample sizes. However, due to the non-random nature of SEER, some limitations of the current study deserved comment. First, in the case of a large population size data, it was not feasible to review the individual pathological diagnosis. Due to the heterogeneity of pathological information and the diversity of pathologist interpretations, there may be misclassifications of SRCC and MC in the SEER database. Because tissue samples were not available, we were unable to determine the histological type of each patient in this study. In order to explore possible heterogeneity between registries, we compared the percentages of SRCC and MC between each center and found no significant differences. Secondly, information regarding chemotherapeutic treatment of patients was not available in SEER data set. Furthermore, information on indicators which might be related with patient prognosis (such as HNPCC, MSI, SAC, CIMP, etc.) has not been recorded in our study. Finally, data on recurrence time and patterns were unavailable in this research. CSS is a reasonable surrogate of CRC-specific outcome.

In conclusion, SRCC had the worst survival among the three histological subtypes of CRC in our study. MC was associated with favorable prognosis in right-sided colon cancer but not in other tumor locations.

## Data Availability

Publicly available datasets were analyzed in this study. This data can be found here: http://seer.cancer.gov/about/overview.html, and the reference number 10058-Nov2013.
