# Supplementary material for: Clinicopathological Features and Survival of Signet-Ring Cell Carcinoma and Mucinous Adenocarcinoma of Right Colon, Left Colon, and Rectum
Source: Pathol Oncol Res. 2021 Jul 2;27:1609800. doi: 10.3389/pore.2021.1609800 (PMC8283508; doi:10.3389/pore.2021.1609800)
Supplement: Supplementary file 1 [file Table1.DOCX]

**TABLE 1.** Clinical and pathological characteristics of colorectal SRCC, MC and NMC patients

| Characteristics | SRCC | MC | NMC | *P* value | *P* value | *P* value |
| --- | --- | --- | --- | --- | --- | --- |
|  | n=6,467 | n=70,555 | n=741,207 | SRCC vs MC | SRCC vs NMC | MC vs NMC |
| Age (years, Mean ±SD) | 65.21 ±16.53 | 69.49 ±13.55 | 69.21 ±15.10 | <0.001 | <0.001 | <0.001 |
| Gender |  |  |  |  |  |  |
| Female | 3,125 (48.3) | 37,113 (52.6) | 358,615 (48.4) | <0.001 | 0.923 | <0.001 |
| Male | 3,342 (51.7) | 33,442 (47.4) | 382,592 (51.6) |  |  |  |
| Race |  |  |  |  |  |  |
| White | 5,443 (84.4) | 59,975 (85.2) | 615,108 (83.3) | <0.001 | 0.021 | <0.001 |
| Black | 560 (8.7) | 6,924 (9.8) | 71,612 (9.7) |  |  |  |
| Others | 448 (6.9) | 3,547 (5.0) | 51,319 (7.0) |  |  |  |
| Unknown | 16 | 109 | 3,168 |  |  |  |
| Year of Diagnosis |  |  |  |  |  |  |
| 1973-1990 | 530 (8.2) | 16,789 (23.8) | 191,047 (25.8) | <0.001 | <0.001 | <0.001 |
| 1991-2000 | 1,740 (26.9) | 19,112 (27.1) | 176,885 (23.9) |  |  |  |
| 2001-2011 | 4,197 (64.9) | 34,654 (49.1) | 373,275 (50.3) |  |  |  |
| Tumor location |  |  |  |  |  |  |
| Right Colon | 3,593 (57.9) | 40,111 (58.4) | 270,070 (37.6) | <0.001 | <0.001 | <0.001 |
| Left Colon | 1,200 (19.4) | 15,775 (23.0) | 233,964 (32.6) |  |  |  |
| Colon^a^ | 5,058 | 57,772 | 527,451 |  |  |  |
| Rectum | 1,409 (22.7) | 12,783 (18.6) | 213,756 (29.8) |  |  |  |
| Tumor numbers |  |  |  |  |  |  |
| Single | 4,649 (71.9) | 48,244 (68.4) | 516,664 (69.7) | <0.001 | <0.001 | <0.001 |
| Multiple | 1,818 (28.1) | 22,310 (31.6) | 224,492 (30.3) |  |  |  |
| Unknown | 0 | 1 | 51 |  |  |  |
| Tumor size (cm) |  |  |  |  |  |  |
| ≤ 5 | 2,258 (49.4) | 25,658 (52.3) | 296,888 (70.9) | <0.001 | <0.001 | <0.001 |
| > 5 | 2,316 (50.6) | 23,447 (47.7) | 121,881 (29.1) |  |  |  |
| Unknown | 1,893 | 21,450 | 322,438 |  |  |  |
| TNM stage^b^ |  |  |  |  |  |  |
| I | 354 (6.1) | 7,547 (13.7) | 140,854 (28.3) | <0.001 | <0.001 | <0.001 |
| II | 975 (16.9) | 19,775 (35.7) | 139,689 (28.0) |  |  |  |
| III | 2,403 (41.7) | 17,174 (31.0) | 124,352 (24.9) |  |  |  |
| IV | 2,034 (35.3) | 10,863 (19.6) | 93,679 (18.8) |  |  |  |
| Unknown | 701 | 15,196 | 242,633 |  |  |  |
| Tumor grade^c^ |  |  |  |  |  |  |
| Well | 46 (0.9) | 7,246 (12.5) | 73,867 (12.7) | <0.001 | <0.001 | <0.001 |
| Moderately | 353 (6.8) | 36,891 (63.9) | 395,133 (68.1) |  |  |  |
| Poorly | 4,363 (83.8) | 12,645 (21.9) | 104,330 (18.0) |  |  |  |
| Undifferentiated | 443 (8.5) | 957 (1.7) | 6,822 (1.2) |  |  |  |
| Unknown | 1,262 | 12,816 | 161,055 |  |  |  |
| Cancer-directed surgery |  |  |  |  |  |  |
| No | 1,114 (17.5) | 3,971 (5.7) | 75,270 (10.5) | <0.001 | <0.001 | <0.001 |
| Yes | 5,263 (82.5) | 65,238 (94.3) | 642,064 (89.5) |  |  |  |
| Unknown | 90 | 1,346 | 23,873 |  |  |  |
| NO. of lymph nodes examined |  |  |  |  |  |  |
| <12 | 3,061 (53.0) | 27,533 (50.3) | 349,246 (61.8) | <0.001 | <0.001 | <0.001 |
| ≥12 | 2,719 (47.0) | 27,255 (49.7) | 215,654 (38.2) |  |  |  |
| Unknown | 687 | 15,767 | 176,307 |  |  |  |
| Radiotherapy |  |  |  |  |  |  |
| No | 5,418 (85.0) | 62,473 (89.7) | 654,061 (89.6) | <0.001 | <0.001 | 0.276 |
| Yes | 957 (15.0) | 7,187 (10.3) | 76,321 (10.4) |  |  |  |
| Unknown | 110 | 895 | 10,825 |  |  |  |

a Colon included large intestinal (188 189 260) Colon vs rectum: *P* <0.001, *P* <0.001 and *P* <0.001 when SRCC vs MC, SRCC vs NMC and MC vs NMC, respectively.

b Stage I+II vs III+IV: *P* <0.001, *P* <0.001 and *P* <0.001 when SRCC vs MC, SRCC vs NMC and MC vs NMC, respectively.

c Well +Moderately vs Poorly +Undifferentiated: *P* <0.001, *P* <0.001 and *P* <0.001 when SRCC vs MC, SRCC vs NMC and MC vs NMC, respectively.
