# Supplementary material for: Clinicopathological Features and Survival of Signet-Ring Cell Carcinoma and Mucinous Adenocarcinoma of Right Colon, Left Colon, and Rectum
Source: Pathol Oncol Res. 2021 Jul 2;27:1609800. doi: 10.3389/pore.2021.1609800 (PMC8283508; doi:10.3389/pore.2021.1609800)
Supplement: Supplementary file 2 [file Table2.DOCX]

**TABLE 2.** The proportion of NMC, MC, SRCC in colorectal cancer in time course

|  | |  | | 1973-1990 | | | | 1991-2000 | | | | 2001-2011 | | | |
| --- | --- | --- | --- | --- | --- | --- | --- | --- | --- | --- | --- | --- | --- | --- | --- |
|  | |  | | Cases | | Constituent ratio (%) | | Cases | | | Constituent ratio (%) | Cases | | Constituent ratio (%) | |
|  | | NMC | | 191047 | | 91.69 | | 176885 | 89.45 | | | 373275 | | 90.57 | |
| All | | MC | | 16789 | | 8.06 | | 19112 | 9.67 | | | 34654 | | 8.41 | |
|  | | SRCC | | 530 | | 0.25 | | 1740 | 0.88 | | | 4197 | | 1.02 | |
|  | | NMC | | 61126 | | 87.19 | | 64070 | 84.20 | | | 144874 | | 86.45 | |
| Right Colon | | MC | | 8745 | | 12.48 | | 11017 | 14.48 | | | 20349 | | 12.14 | |
|  | | SRCC | | 234 | | 0.33 | | 1007 | 1.32 | | | 2352 | | 1.41 | |
|  | | NMC | | 66130 | | 93.67 | | 57042 | 92.60 | | | 110792 | | 93.31 | |
| Left Colon | | MC | | 4338 | | 6.14 | | 4246 | 6.89 | | | 7191 | | 6.06 | |
|  | | SRCC | | 133 | | 0.19 | | 312 | 0.51 | | | 755 | | 0.63 | |
|  | | NMC | | 57524 | | 94.26 | | 50193 | 93.03 | | | 106039 | | 93.87 | |
| Rectum | | MC | | 3361 | | 5.51 | | 3407 | 6.31 | | | 6015 | | 5.32 | |
|  | | SRCC | | 142 | | 0.23 | | 355 | 0.66 | | | 912 | | 0.81 | |
