# Supplementary material for: Clinicopathological Features and Survival of Signet-Ring Cell Carcinoma and Mucinous Adenocarcinoma of Right Colon, Left Colon, and Rectum
Source: Pathol Oncol Res. 2021 Jul 2;27:1609800. doi: 10.3389/pore.2021.1609800 (PMC8283508; doi:10.3389/pore.2021.1609800)
Supplement: Supplementary file 3 [file Table3.DOCX]

**TABLE 3.** The 5-year cancer-specific survival for SRCC, MC and NMC patients with AJCC stages I, II, III, and IV according to tumor location

|  | Age groups | (% stand error) |  | *P* value |  |  |
| --- | --- | --- | --- | --- | --- | --- |
|  | SRCC | MC | NMC | SRCC vs MC | SRCC vs NMC | MC vs NMC |
| **Right Colon** |  |  |  |  |  |  |
| All stages | 35.4 (1.04%) | 64.6 (0.298%) | 62.8 (0.116%) | <0.001 | <0.001 | 0.010 |
| I | 77.71 (4.52%) | 91.3 (0.561%) | 90.2 (0.186%) | <0.001 | <0.001 | 0.088 |
| II | 74.47 (2.19%) | 83.4 (0.419%) | 82.0 (0.194%) | <0.001 | <0.001 | 0.001 |
| III | 38.64 (1.67%) | 59.3 (0.614%) | 58.8 (0.279%) | <0.001 | <0.001 | 0.996 |
| IV | 3.64 (0.825%) | 10.0 (0.514%) | 7.38 (0.190%) | <0.001 | 0.016 | <0.001 |
| **Left Colon** |  |  |  |  |  |  |
| All stages | 25.2 (1.6%) | 53.7 (0.478%) | 68.2 (0.116%) | <0.001 | <0.001 | <0.001 |
| I | 85.3 (6.1%) | 91.9 (0.893%) | 93.6 (0.141%) | 0.141 | 0.042 | 0.056 |
| II | 60.77 (5.86%) | 76.9 (0.821%) | 78.9 (0.249%) | <0.001 | <0.001 | 0.005 |
| III | 35.39 (3.09%) | 57.4 (1.04%) | 66.5 (0.307%) | <0.001 | <0.001 | <0.001 |
| IV | 3.97 (1.20%) | 9.06 (0.684%) | 11.1 (0.252%) | <0.001 | <0.001 | <0.001 |
| **Rectum** |  |  |  |  |  |  |
| All stages | 25.05 (1.44%) | 49.3 (0.531%) | 63.2 (0.126%) | <0.001 | <0.001 | <0.001 |
| I | 55.78 (6.18%) | 80.56 (1.26%) | 87.1 (0.194%) | <0.001 | <0.001 | <0.001 |
| II | 33.60 (4.49%) | 66.28 (1.15%) | 73.1 (0.304%) | <0.001 | <0.001 | <0.001 |
| III | 34.55 (2.56%) | 53.9 (1.03%) | 63.1 (0.321%) | <0.001 | <0.001 | <0.001 |
| IV | 2.46 (1.03%) | 10.6 (0.914%) | 10.1 (0.257%) | <0.001 | <0.001 | 0.757 |
