# Supplementary material for: Clinicopathological Features and Survival of Signet-Ring Cell Carcinoma and Mucinous Adenocarcinoma of Right Colon, Left Colon, and Rectum
Source: Pathol Oncol Res. 2021 Jul 2;27:1609800. doi: 10.3389/pore.2021.1609800 (PMC8283508; doi:10.3389/pore.2021.1609800)
Supplement: Supplementary file 4 [file Table4.DOCX]

**TABLE 4.** Multivariate survival analysis of prognostic factors in colon and rectal patients

|  | Right Colon |  |  | Left Colon |  |  | Rectum |  |  |
| --- | --- | --- | --- | --- | --- | --- | --- | --- | --- |
| Variable | HR | 95% CI | *P* value | HR | 95% CI | *P* value | HR | 95% CI | *P* value |
| Histologic type |  |  |  |  |  |  |  |  |  |
| NMC | 1 |  |  | 1 |  |  | 1 |  |  |
| MC | 0.961 | 0.936 to 0.987 | 0.003 | 1.160 | 1.115 to 1.207 | <0.001 | 1.220 | 1.167 to 1.276 | <0.001 |
| SRCC | 1.225 | 1.148 to 1.308 | <0.001 | 1.593 | 1.426 to 1.779 | <0.001 | 1.774 | 1.582 to 1.988 | <0.001 |
| Gender |  |  |  |  |  |  |  |  |  |
| Female | 1 |  |  | 1 |  |  | 1 |  |  |
| Male | 1.059 | 1.040 to 1.079 | <0.001 | 1.043 | 1.019 to 1.067 | <0.001 | 1.054 | 1.029 to 1.080 | <0.001 |
| Age (years) |  |  |  |  |  |  |  |  |  |
| <mean 70 | 1 |  |  | 1 |  |  | 1 |  |  |
| ≥mean 70 | 1.574 | 1.544 to 1.605 | <0.001 | 1.758 | 1.718 to 1.799 | <0.001 | 1.736 | 1.693 to 1.780 | <0.001 |
| Race |  |  |  |  |  |  |  |  |  |
| White | 1 |  |  | 1 |  |  | 1 |  |  |
| Black | 1.149 | 1.117 to 1.182 | <0.001 | 1.256 | 1.213 to 1.299 | <0.001 | 1.307 | 1.253 to 1.363 | <0.001 |
| Others | 0.884 | 0.850 to 0.920 | <0.001 | 0.886 | 0.851 to 0.921 | <0.001 | 0.919 | 0.882 to 0.959 | <0.001 |
| Year at diagnosis |  |  |  |  |  |  |  |  |  |
| 1973-1990 | 1 |  |  | 1 |  |  | 1 |  |  |
| 1991-2000 | 0.915 | 0.880 to 0.952 | <0.001 | 0.887 | 0.849 to 0.928 | <0.001 | 0.857 | 0.819 to 0.897 | <0.001 |
| 2001-2011 | 0.823 | 0.792 to 0.856 | <0.001 | 0.766 | 0.733 to 0.801 | <0.001 | 0.672 | 0.642 to 0.703 | <0.001 |
| Tumor numbers |  |  |  |  |  |  |  |  |  |
| Single | 1 |  |  | 1 |  |  | 1 |  |  |
| Multiple | 0.632 | 0.613 to 0.652 | <0.001 | 0.644 | 0.621 to 0.669 | <0.001 | 0.609 | 0.585 to 0.635 | <0.001 |
| Tumor size (cm) |  |  |  |  |  |  |  |  |  |
| ≤ 5 | 1 |  |  | 1 |  |  | 1 |  |  |
| > 5 | 1.116 | 1.095 to 1.137 | <0.001 | 1.179 | 1.150 to 1.208 | <0.001 | 1.249 | 1.217 to 1.282 | <0.001 |
| TNM stage |  |  |  |  |  |  |  |  |  |
| I | 1 |  |  | 1 |  |  | 1 |  |  |
| II | 1.946 | 1.855 to 2.040 | <0.001 | 2.529 | 2.389 to 2.677 | <0.001 | 1.995 | 1.907 to 2.088 | <0.001 |
| III | 5.027 | 4.802 to 5.263 | <0.001 | 4.771 | 4.514 to 5.042 | <0.001 | 3.500 | 3.353 to 3.653 | <0.001 |
| IV | 21.793 | 20.803 to 22.830 | <0.001 | 21.573 | 20.402 to 22.811 | <0.001 | 13.198 | 12.617 to 13.805 | <0.001 |
| Tumor grade |  |  |  |  |  |  |  |  |  |
| Well | 1 |  |  | 1 |  |  | 1 |  |  |
| Moderately | 1.122 | 1.076 to 1.171 | <0.001 | 1.136 | 1.084 to 1.191 | <0.001 | 1.095 | 1.041 to 1.152 | <0.001 |
| Poorly | 1.518 | 1.452 to 1.587 | <0.001 | 1.542 | 1.464 to 1.625 | <0.001 | 1.566 | 1.481 to 1.655 | <0.001 |
| Undifferentiated | 1.606 | 1.486 to 1.735 | <0.001 | 1.724 | 1.527 to 1.945 | <0.001 | 1.895 | 1.666 to 2.156 | <0.001 |
| Cancer-directed surgery |  |  |  |  |  |  |  |  |  |
| No | 1 |  |  | 1 |  |  | 1 |  |  |
| Yes | 0.468 | 0.444 to 0.493 | <0.001 | 0.432 | 0.406 to 0.459 | <0.001 | 0.404 | 0.387 to 0.422 | <0.001 |
| NO. of lymph nodes examined |  |  |  |  |  |  |  |  |  |
| <12 | 1 |  |  | 1 |  |  | 1 |  |  |
| ≥12 | 0.733 | 0.718 to 0.747 | <0.001 | 0.770 | 0.751 to 0.788 | <0.001 | 0.816 | 0.795 to 0.839 | <0.001 |
| Radiotherapy |  |  |  |  |  |  |  |  |  |
| No | 1 |  |  | 1 |  |  | 1 |  |  |
| Yes | / | / | / | / | / | / | 0.843 | 0.821 to 0.865 | <0.001 |
